# Imported Fatal Hantavirus Pulmonary Syndrome

**DOI:** 10.3201/eid1309.070708

**Published:** 2007-09

**Authors:** Steven Reynolds, Eleni Galanis, Mel Krajden, Muhammad Morshed, David Bowering, William Abelson, Tobias R. Kollmann

**Affiliations:** * University of British Columbia, Vancouver, British Columbia, Canada; † British Columbia Center for Disease Control, Vancouver, British Columbia, Canada; ‡ Office of Chief Medical Officer, Northern Health Region, British Columbia, Vancouver, British Columbia, Canada

**Keywords:** Hantavirus infections, Bolivia, British Columbia, Canada, infection control, letter

**To the Editor:** Hantavirus pulmonary syndrome (HPS) is characterized by fever, gastrointestinal symptoms, respiratory distress, elevated hematocrit, hypoalbuminemia, and thrombocytopenia. Most cases in North America are acquired from rodent vectors and are caused by the Sin Nombre virus. Person-to-person transmission has been reported for Andes virus (*1**,**2*) but not for Sin Nombre virus (*3*). We describe a patient with fatal hantavirus pulmonary syndrome.

The patient was a previously healthy 15-year-old Canadian girl. In the spring of 2006, she had traveled to the Santa Cruz-San Jose de Chiquitas corridor of Bolivia with her parents and siblings for a 4-week visit (Figure), where they stayed with family and friends on their farms. The family noted rodent droppings outside but no rodents were seen. The patient had no known exposure to rodents or rodent droppings after her return to Canada.

**Figure:**
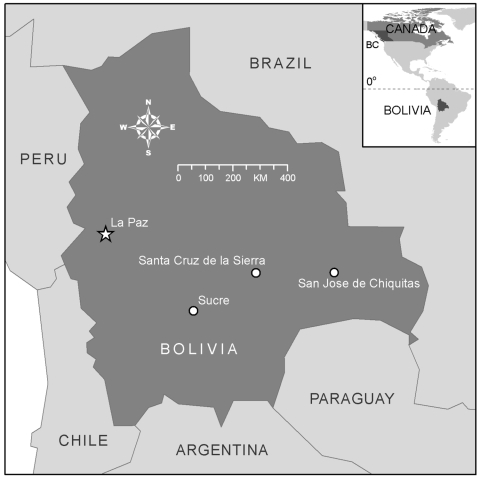
Map of Bolivia with an inset map of North America showing the location of British Columbia (BC) and its relation to Bolivia.

On day 26 after her return from Bolivia, the patient sought treatment at a community hospital at 6:30 am for malaise and mild fever. During the night before seeking treatment, she had mild confusion. Her initial blood pressure was 99/50, heart rate 97, and oxygen saturation 96% on room air. Her hemoglobin was 192 g/L (reference range 117–149), platelets 82 × 10^9^/L (reference range 165–400), and leukocyte count 7.5 × 10^9^/L (reference range 3.9–10.2). She was initially treated with 3 liters of normal saline and repeat hemoglobin tests showed a value of 206 g/L. Due to ongoing hypotension and hypoxia, she was intubated and sedated. Rocuronium was used as a paralytic agent to facilititate high pressure mechanical ventilation and maintain patient-ventilator synchrony. Dopamine and epinephrine were given as intravenous drips. Arrangements were made for the patient to be transferred to a tertiary pediatric care center for possible extracorporeal membrane oxygenation. During air transport, she had an asystolic cardiac arrest. While administering cardiopulmonary resuscitation, members of the healthcare team were exposed to a considerable volume of pulmonary edema fluid expelled from the patient’s endotracheal tube. Few, if any, were able to maintain adequate protection with face shields or protective eyewear. Resuscitation efforts were unsuccessful, and the patient was pronounced dead on arrival at the tertiary care center at 7:26 pm. Postmortem examination showed evidence of marked pulmonary edema, diffuse alveolar damage, and lymphoid inflammation in the pulmonary interstitium.

Serologic examination of an acute blood sample was immunoglobulin M (IgM) positive for Sin Nombre virus, but low optical densities indicated potential for an infection with a related hantavirus rather than Sin Nombre virus. Subsequently, reverse transcription–PCR (RT-PCR) on blood in EDTA and lung tissue followed by sequence analysis confirmed an Andes-like hantavirus infection. None of the 40 household and healthcare contacts of the patient had symptoms compatible with HPS during an 8-week monitoring period. Two contacts with nonspecific symptoms were tested and found to be negative for hantavirus-specific immunoglobulin (Ig) M and IgG and negative by RT-PCR.

Additonally, a seroprevalence survey of close contacts and assessment of level of contact was conducted. Close contacts were defined as persons who lived in the same household as the patient, were in the same enclosed space for >2 hours, or provided healthcare to her while she was symptomatic. Twenty-eight (62%) of 45 close contacts provided serum over the next 5 months. All serum samples were negative for Sin Nombre and Andes IgG and Sin Nombre IgM by ELISA. Fourteen (50%) of the 28 completed a self-administered questionnaire which assessed the type and intensity of contact. Of these, 12 were healthcare workers and 2 were friends. One friend had contact with the patient 3 days before she died, a friend and a healthcare worker had contact with her on the day before her death, and the rest of the healthcare workers had contact with the patient on the day she died.

To our knowledge, this is the first imported case and the tenth case of HPS reported in British Columbia, Canada, since 1994 (2005 BC Annual Summary of Reportable Diseases at www.bccdc.org/content.php?item=33) (*4*). Six of these 10 cases were fatal. All cases except the 1 described here have been locally acquired Sin Nombre infections. Sin Nombre virus is endemic in the *Peromyscus maniculatus* (deer mice) population in most of British Columbia (*5*).

Worldwide, imported cases of HPS are unusual, although HPS has been reported in countries that are in close geographic proximity or in travelers to disease-endemic areas (*6**–**8*). Fortunately, none of the persons exposed to the patient reported symptoms consistent with HPS during the incubation period, and none who were tested seroconverted. Seroprevalence surveys in Chile among healthcare worker contacts of patients with HPS caused by the Andes virus showed a prevalence of 0% (*9*). A report from Argentina showed that cases due to secondary transmission occurred mostly in non-healthcare workers after prolonged close contact in the prodromal period (*10*). In conclusion, we describe an imported case of fatal HPS due to an Andes-like hantavirus with no evidence of secondary transmission.
